# Retinoic acid treatment of human leiomyoma cells transformed the cell phenotype to one strongly resembling myometrial cells

**DOI:** 10.1111/j.1365-2265.2008.03207.x

**Published:** 2008-09

**Authors:** Minnie Malik, Joy Webb, William H Catherino

**Affiliations:** * Department of Obstetrics and Gynecology, Uniformed Services University of the Health SciencesBethesda, MD, USA; † Reproductive Biology and Medicine Branch, National Institute of Child Health and Human Development, NIHBethesda, MD, USA

## Abstract

**Background:**

Uterine leiomyomas are clinically significant tumours that may develop due to an altered differentiation pathway. We have previously identified a dysregulated retinoic acid (RA) pathway that reduced retinoic exposure in human leiomyoma surgical specimens, and have shown that the leiomyoma phenotype was characterized by excessive and disorganized extracellular matrix (ECM).

**Objective:**

The goal of this study was to determine the impact of RA exposure on the disrupted ECM phenotype of leiomyomas.

**Design and methods:**

Study of immortalized and molecularly confirmed cells generated from surgical specimens of spontaneous uterine leiomyoma and matched myometrium.

**Results:**

Immortalized leiomyoma and myometrial cells retained the molecular characteristics of their progenitor tissue. Proliferation of leiomyoma cells was inhibited by all*-trans* retinoic acid (ATRA). Furthermore, there was a dose-dependent decrease in soluble extracellular collagen protein in ATRA-treated leiomyoma cells. Exposure of leiomyoma cells to ATRA resulted in a dose-dependent inhibition of templates for specific ECM protein production including collagen 1, collagen 4, fibronectin and versican. Notably, expression levels in treated leiomyoma cells approached those found in myometrial cells. These mRNA alterations translated into altered protein. Down-regulation was also observed among the RA pathway genes such as CYP26A1 with exposure to ATRA. Finally, ATRA down-regulated TGF-β3 mRNA expression and the TGF-β regulated genes in leiomyoma cells.

**Conclusion:**

Exposure of leiomyomas to ATRA down-regulated cell proliferation, ECM formation, RA metabolism and TGF-β regulation, suggesting that RA exposure can alter the leiomyoma phenotype to one that more closely approximates normal myometrium.

## Introduction

Uterine leiomyomas impact 20%–40% of women over 35 years of age in United States.^1^ Enlargement of these tumours can cause pelvic pain, menorrhagia, reduced fertility, pregnancy loss and a number of other serious gynaecological problems. Women with symptomatic leiomyoma may be obligated to undergo hysterectomy as it is the only definitive treatment for this disease. As hysterectomy precludes future childbirth, novel interventions are warranted to treat these tumours.

Leiomyomas are oestrogen and progesterone responsive,^2^ but gonadal hormones only promote growth, as GnRH agonist-induced hypogonadism does not eliminate these tumours and the tumours rapidly re-grow when gonadal hormone exposure resumes. Recently, we have hypothesized that leiomyomas are a disease of differentiation^3^ leading to a disrupted extracellular matrix (ECM). Altered differentiation patterns in various normal tissues including keratinocytes and neurones, as well as neoplasms such as leukaemia, ovarian carcinoma and endometrial adenocarcinoma are known to be influenced by retinoids.^4^,^5^ We have recently demonstrated that genes involved in retinoic acid (RA) production were reduced in leiomyoma surgical specimens, while the RA metabolizing genes were increased, resulting in a reduced amount of active RA.^6^ We also demonstrated that the rate of RA metabolism is higher in leiomyoma compared to myometrium tissue.^6^ Based on these findings, we hypothesized that reduced intracellular all*-trans-*retinoic acid (ATRA) concentrations in leiomyoma cells may result in alteration of various signalling pathways that control its phenotype. Central to the leiomyoma phenotype is the over-expression of ECM.^7^ In this study, we determined whether exposure to ATRA impacts expression of ECM genes and central signalling pathways in leiomyoma.

## Materials and methods

### Immortalization of cell cultures

After obtaining IRB approval and consent from patients undergoing medically indicated hysterectomy for symptomatic leiomyomas, both myometrium and leiomyoma tissue were harvested at the National Naval Medical Center, Bethesda, MD. The collection of tissue and generation of primary cultures has been described previously.^8^ The primary cultures of myometrium and leiomyoma were immortalized using HPV-16 genes as described^9^ with minor modifications. Briefly, the primary cultures from first passage were allowed to grow 40%–50% confluence before infection with retrovirus stock (pSLXN virus with genticin selection gene was a gift from Dr Rhim, Center for Prostrate Disease Research, Bethesda, MD). Polybrene (5 µg/ml) was added to each flask to enhance infection by the retroviral vector. After incubation at 37 °C for 24 h, the cells were washed once with PBS, heated to 37 °C and cultured in fresh DMEM-F12 supplemented with 10% foetal bovine serum (FBS). The cells were maintained at 37 °C and 5% CO_2_ for 48 h before fresh media containing 100 µg/ml of genticin (G418, Sigma-Aldrich, St Louis, MO) was added. The cells were grown for 4 days in selection media before fresh media was added. The immortalized cells proliferated to confluency before trypsinization and further maintenance of the cells. Compared to primary cultures that senesced at passage 10 and were no longer viable by passage 12, the immortalized cells were growing well past passage 25.

### Confirmation of E6 gene expression

To confirm that the cells expressed HPV-16 E6 gene, end-point reverse transcriptase polymerase chain reaction (RT-PCR) was performed. RNA was isolated from both myometrial and leiomyoma immortalized cells cultures using the TRIzol Reagent (Invitrogen, Carlsbad, CA) according to manufacturer's protocol and was described previously.^6^,^8^,^10^

### Confirmation of cell cultures

The immortalized cell lines were confirmed to be derived either from myometrium or leiomyoma using the specific biomarker gene array as described previously.^8^ Using real-time RT-PCR, expression of the genes dermatopontin, versican, TGF-β3 and CYP26A1 were analysed in the cell cultures.

### Cell proliferation studies

Myometrial (1 × 10^3^ cells/well) and leiomyoma cells (1·5 × 10^3^ cells/well) were plated in 96-well plates, and allowed to grow for 2 days before being exposed to graded ATRA concentrations. Two plates for each cell line was collected every 24 h for up to 96 h. The proliferation of the cells was measured using sulphorhodamine-B method (Sigma-Aldrich) according to manufacturer's protocol. Data represents three separate culture studies with six replicates of each concentration per plate.

### ATRA exposure

Immortalized myometrial and leiomyoma cells were plated at densities of approximately 2 × 10^4^ cells/well in 6-well plates and allowed to grow till 60% confluency before exposure to ATRA at 10^−8^m to 10^−5^m, as well as the vehicle-only control. The experiment was repeated three times with duplicates at each concentration. After 24 h exposure to ATRA, the cells were lysed and RNA and protein collected for further analysis. The media was collected, supplemented with 1× protease inhibitor (Pierce Biotechnology, Rockford, IL) before storage at –80 °C for collagen analysis.

### Soluble collagen expression

To determine soluble collagen, we used the SIRCOL method (Bicolor, Accurate Chemical and Scientific Corp., Westbury, NY) according to manufacturer's recommendation, with modifications. The collection and storage of the media has been described above. On the day of the experiment the media was thawed and collagen precipitated by addition of 4 m NaCl. After gentle rocking at 4 °C overnight, the tubes were centrifuged at 20 000 ***g*** for 40 min to pellet the collagens. To generate a standard curve, known amounts of collagen (Type 1; provided in the kit) underwent a similar precipitation procedure. The pellet was dissolved in 0·5 m acetic acid; the volume was the same as the volume of starting media. Aliquots of 100 µl for each sample were taken in triplicate and Sircol dye reagent added. The tubes were allowed to gently mix for 30 min at room temperature before centrifugation at 15 000 ***g*** for 10 min The collagen-dye pellet was dissolved in alkali reagent supplied with the kit, and measured at 540 nm using Benchmark plus (Bio-Rad, Hercules, CA).

### Reverse transcriptase polymerase chain reaction (RT-PCR)

End-point and real-time RT-PCR methods have been described previously.^6^,^8^,^10^ Briefly, the adherent cells were lysed using TRIzol, and chloroform (0·2 ml per ml of TRIzol) was added. The samples were centrifuged at 12 000 ***g*** for 15 min at 4 °C. To the aqueous phase ice-cold isopropanol was added at 0·5 ml per ml TRIzol to precipitate RNA. The RNA pellet was washed once with 70% DEPC–ethanol, briefly dried and dissolved in DEPC-treated water. To ensure that there was no contaminating DNA present, all RNA samples were treated with DNAse-I enzyme using the DNA-free kit in accordance to the manufacturer's protocol (Ambion, Austin, TX). The RNA was quantified spectrophometrically before storage at –80 °C.

For real-time RT-PCR, primers were synthesized in-house by Biomedical Instrumentation Center and the probes were synthesized by Integrated DNA Technologies as described previously.^8^ Real-time RT-PCR was done using iScript kit (Bio-Rad) and Bio-Rad iCycler software was used for data analysis.

### Cytoimmunofluorescence

The method used was described previously with some modifications.^8^ Briefly, the cells were grown on glass chamber slides and treated with ATRA (10^−7^m) for 24 h. The cells were fixed with cold methanol and permeabilized with 0·2% Triton-X100 (Sigma-Aldrich). Nonspecific sites were blocked using blocking buffer (1% BSA + 10% normal goat serum) before addition of primary antibody (fibronectin, Santa Cruz Biotechnology, Santa Cruz, CA). After overnight incubation at 4 °C the cells were washed with wash solution (PBS + 0·05% Triton + 1% BSA). Fluorescent secondary antibody conjugated with Alexa-594 (Invitrogen) was used at recommended concentration (1 µg/ml). The mounting media (Vector Laboratories, Burlingame, CA) contains the nucleic acid binding dye DAPI. The slides were examined with an Axiovert 405M epifluorescence inverted light microscope (Carl Zeiss, Thornwood, NJ). Images were acquired with a CCD camera (Orca-ER, Hamamatsu City, Japan).

### Western blot

Protein was isolated using M-PER (Pierce Biotechnology) according to manufacturer's protocol. Protein was measured and stored at –80 °C. The methodology has been described previously.^6^ Briefly, protein samples were denatured in 1× SDS buffer before electrophoresis on SDS-PAGE gels (Invitrogen) and the separated proteins were electro-blotted to nitrocellulose membrane. Versican V0/V1 Neo primary antibody (Affinity Bioreagents, Golden, CO) was used and detected with the horseradish peroxidase (HRP)-conjugated secondary antibody (ImmunoPure, Pierce Biotechnology) in combination with the SuperSignal West Pico (Pierce Biotechnology). As an internal standard between the samples, antihuman β-actin (Santa Cruz Biotechnology) at a dilution of 1 : 50 000 was used.

### Statistical analysis

All experiments were repeated a minimum of three times. For real-time RT-PCR data, the results are reported as mean ± SEM. For each result the average expression of three replicates was calculated before relative quantification using normalization against housekeeping gene (18S) was done. Relative expression was calculated based on Pfaffl method.^11^ Wilcoxon signed rank test was used for nonparametric statistical evaluation. For proliferation data, statistical significance was calculated by anova or student's *t*-test. Values below *P* < 0·05 were considered significant. For Western blot analysis, calculations were done using QualityOne software from Bio-Rad. Data is presented as fold difference between leiomyoma and myometrium (L : M) relative density units that was corrected for internal control, β-actin.

## Results

### Confirmation of Immortalized cell cultures

We developed immortalized cell cultures of leiomyoma and patient matched myometrium by stably transfecting and expressing HPV-16 E6/E7 genes as described previously.^9^ The E6 gene specific product was observed in immortalized myometrial and leiomyoma cells (data not shown). The RNA isolated from nontransfected primary cultures did not demonstrate E6 gene product.

One of the most significant challenges of primary cultures is the loss of oestrogen receptor and progesterone receptor expression with late passages. We therefore confirmed that our immortalized cell lines express oestrogen receptor and progesterone receptor in both early and late passages (passages 7 and 14; data not shown).

We further validated leiomyoma from myometrial cultures based on the molecular characteristics that distinguish leiomyoma from the normal myometrium.^8^ The expression of dermatopontin was markedly reduced in immortalized leiomyoma cells compared to patient matched myometrial cells (–15·2 ± 3·2-fold, *P* < 0·05). Versican (3·05 ± 0·79-fold, *P* < 0·05), CYP26A1 (19·16 ± 3·98-fold, *P* < 0·05) and TGF-β3 (2·6 ± 1·02-fold, *P* < 0·05) demonstrated increased gene expression in leiomyoma cells, similar to the primary cultures and progenitor tissue.^8^ Changes in expression of these genes were maintained throughout multiple cell passages for both leiomyoma and myometrial cell cultures (data not shown).

### Cell proliferation inhibited in response to ATRA exposure

**Fig. 1:**
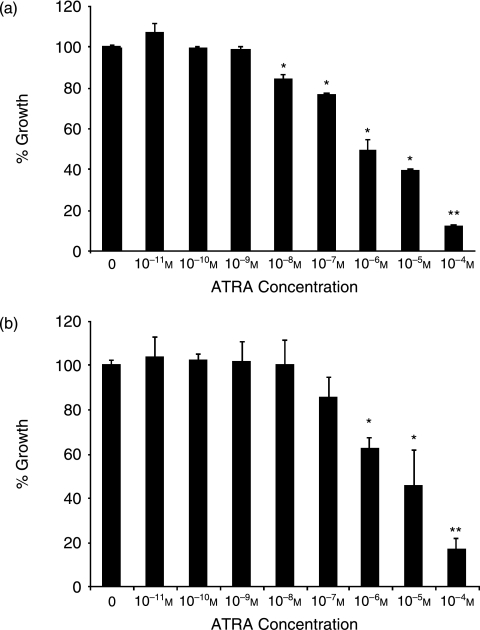
Leiomyoma and myometrial cells demonstrated ATRA concentration-dependent growth inhibition. Compared to control unexposed cells, significant growth inhibition was observed at 10^−8^m and above in leiomyoma (a) and at 10^−6^m and above in myometrial cells (b).

Leiomyoma and myometrial cells treated with ATRA demonstrated an inhibition of growth compared to untreated cells (Fig. 1). Statistically significant growth inhibition of leiomyoma cells occurred at 10^−8^m ATRA (Fig. 1a). Further growth inhibition was observed at higher concentrations of ATRA. Myometrial cells demonstrated significant growth inhibition at 10^−6^m concentration (Fig. 1b). These results suggest a greater sensitivity of leiomyoma cells to ATRA compared with myometrial cells, resulting in growth inhibition at 10^−8^m. This finding was unexpected, given the greater ability of leiomyoma cells to metabolize ATRA to inactive compounds.^6^ We therefore investigated the impact of ATRA treatment on expression of genes involved in RA metabolism.

### RA pathway genes regulated by ATRA treatment

RA regulates a number of genes involved in the RA pathway including the receptors and metabolizing enzymes such as CYP26.^12^–^14^ Up-regulation of CYP26A1 expression protects many cell types from apoptosis.^15^ We have previously demonstrated that CYP26 genes were up-regulated in leiomyomas as compared to myometrial tissue.^6^ Untreated leiomyoma cells (0 µm) demonstrated a 16·8 ± 4·5-fold up-regulation (*P* < 0·05) of CYP26A1 gene expression compared with myometrial cells (Table 1). At lower concentrations of ATRA (10^−8^ m and 10^−7^ m), CYP26A1 was down-regulated two to fourfold in leiomyoma cells compared with myometrial cells. At 10^−6^ m and higher concentrations, the fold difference of expression was similar in both leiomyoma and myometrial cell lines (0·91 ± 0·02; *P* < 0·05).

**Table 1:** Regulation of leiomyoma : myometrial cell gene expression by retinoic acid

	ATRA concentrations (m)			
	
Genes analyzed	0	10^−8^	10^−7^	10^−6^
RA genes				
CRBP-1	0·38 ± 0·14	0·91 ± 0·13*	1·42 ± 0·19*	1·51 ± 0·38*
CYP26A1	16·81 ± 4·50	4·44 ± 1·46*	2·00 ± 1·08*	0·91 ± 0·02*
ECM genes				
Collagen 1A1	2·72 ± 0·55	0·61 ± 0·14*	0·41 ± 0·04*	0·38 ± 0·18*
Collagen 3A1	1·99 ± 0·39	2·99 ± 1·31	2·50 ± 0·09	2·14 ± 0·29
Collagen 4A1	2·95 ± 0·37	0·89 ± 0·12*	1·13 ± 0·14*	1·33 ± 0·21*
Collagen 7A1	0·51 ± 0·11	0·56 ± 0·01	1·91 ± 0·17*	1·71 ± 0·12*
Fibronectin	2·18 ± 0·12	1·72 ± 0·41	1·62 ± 0·32	1·41 ± 0·23*
Versican V0	7·75 ± 2·2	7·73 ± 0·38	2·94 ± 0·43*	0·96 ± 0·04*
Versican V1	4·6 ± 0·45	0·71 ± 0·13*	0·70 ± 0·06*	0·19 ± 0·13*
Versican V3	8·32 ± 1·21	2·34 ± 0·68*	1·15 ± 0·22*	2·73 ± 0·13*
TGF-β pathway				
TGF-β3	2·96 ± 0·71	0·84 ± 0·12*	0·12 ± 0·01*	0·09 ± 0·02*
MMP2	6·65 ± 1·22	0·56 ± 0·47*	0·36 ± 0·01*	0·67 ± 0·43*

Fold changes (leiomyoma : myometrial cell expression) in mRNA amount indicative of expression of genes that were analyzed in response to ATRA exposure. Experiments were performed in triplicate. Data are presented as mean ± SEM and were analyzed by Wilcoxon matched-pairs signed rank test.

* Significant difference (*P* < 0·05) compared to control (0 m). ATRA, all-*trans*-retinoic acid; RA, retinoic acid; ECM, extracellular matrix; CRBP-1, cellular retinol binding protein-1; TGF-β3, transforming growth factor β3; MMP2, matrix metalloproteinase.

We have previously shown that cellular retinol binding protein (CRBP-1) was down-regulated in leiomyoma tissue as compared to myometrium.^6^ In this study, we have demonstrated that our immortalized cell lines also show a similar threefold down-regulation of CRBP-1 mRNA expression (0·38 ± 0·14; *P* < 0·05) in untreated leiomyoma cells compared to myometrial cells (Table 1). A concentration-dependent up-regulation of the gene was observed on treatment with ATRA (Table 1). With 10^−8^m ATRA treatment, the expression of the CRBP-1 in leiomyoma was similar to myometrial cells.

These results suggest that ATRA exposure at approximately 10^−8^m can overcome the alterations in RA metabolism genes found in leiomyomas, resulting in expression patterns comparable to myometrial cells. Phenotypically, leiomyoma differs from normal myometrium in the expression of excessive disorganized ECM.^3^ We therefore evaluated total collagen expression in leiomyoma cells when treated with ATRA.

### ATRA exposure reduced collagen production

**Fig. 2:**
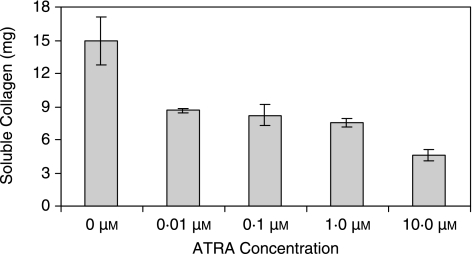
Effect of ATRA on secretion of collagens by leiomyoma cells. Reduced amount of collagens was secreted in the media of leiomyoma cells treated with ATRA. Soluble collagen was measured using the Sircol method. Significant decrease in soluble collagens secreted (9 mg) was measured at 10^−8^ m ATRA as compared to 15 mg in untreated cells (0 m).

Leiomyomas are characterized by excessive collagen production in the ECM.^7^,^16^ We therefore evaluated the effect of ATRA treatment on collagen production in leiomyoma cell cultures by measuring salt soluble collagen secreted into the media after 24 h of ATRA treatment, prior to one cycle of cell division. As shown in Fig. 2, leiomyoma cells demonstrated a decrease in collagen secretion with increasing ATRA exposure. At 10^−8^m ATRA, only 9 mg of soluble collagen was measured in the media as compared to 15 mg in untreated cells.

### ATRA regulated expression of individual ECM components

**Fig. 3:**
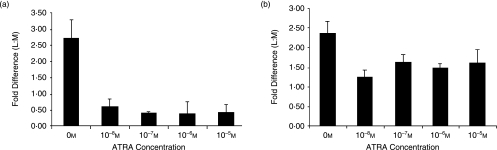
Expression of collagens in leiomyoma and effect of ATRA treatment. (a) Collagen 1A1 fibril template was elevated in leiomyoma (2·72 ± 0·55-fold; *P* < 0·05) as compared to myometrial cells and demonstrated a down-regulation at different concentrations of ATRA. (b) Collagen 4A1 fibril template was elevated in leiomyoma (2·95 ± 0·37-fold; *P* < 0·05) and demonstrated a fold down-regulation at all concentrations of ATRA tested.

With a decrease in soluble collagen secretion, we hypothesized that the expression of individual ECM components known to be disrupted in leiomyomas may also be regulated by ATRA. We selected collagen genes, based on previous work^16^ that are known to be differentially expressed in leiomyomas compared to myometrium. The mRNA expression was increased for collagen genes COL1A1 (2·72 ± 0·55-fold; *P* < 0·05) and COL4A1 (2·95 ± 0·37-fold; *P* < 0·05) in leiomyoma cells as compared to myometrial cells prior to treatment (Fig. 3). With ATRA treatment, a down-regulation of collagen genes was observed. A fourfold down-regulation (0·61 ± 0·14-fold; *P* < 0·05) of COL1A1 gene template was observed in leiomyoma cells at concentration of 10^−8^m ATRA (Fig. 3a), with no further significant fold change at higher concentrations. COL4A1 template also demonstrated a down-regulation of gene expression in treated leiomyoma cells as compared to treated myometrial cells (Fig. 3b) with greatest change at 10^−8^m ATRA (0·89 + 0·12; *P* < 0·05). At higher concentrations of ATRA, the relative COL4A1 gene expression between leiomyoma and myometrial cells remained comparable.

Collagen 7A1 (COL7A1) gene expression was lower (0·51 ± 0·11-fold; *P* < 0·05) in leiomyoma cells in culture as compared to myometrial cells in the absence of ATRA exposure (Table 1). At ATRA concentrations of 10^−7^m, a twofold up-regulation of COL 7A1 gene expression was demonstrated with no further change in expression at higher concentrations (Table 1).

Collagen 3A1 (COL3A1) template demonstrated an up-regulation of gene expression (1·99 ± 0·39; *P* < 0·05) in untreated leiomyomas compared to myometrial cells. We found that mRNA expression of this collagen was unaffected by exposure to ATRA treatment (Table 1).

Taken together, ATRA exposure reduced COL1A1 and COL4A1 mRNA expression in leiomyoma cells, approaching expression patterns comparable to myometrial cells. The expression of COL7A1 template was increased by ATRA exposure, resulting in similar comparable expression. However, not all collagens are altered by ATRA exposure, as demonstrated with COL3A1 template regulation.

### Fibronectin expression in response to ATRA exposure

Fibronectin is an extracellular adhesion protein that binds to its receptors on cell membrane and collagen in the matrix, thus anchoring the cell. As shown in Table 1, untreated leiomyoma cells demonstrated a 2·18 ± 0·12-fold (*P* < 0·05) increase in fibronectin mRNA expression compared to myometrial cells. With ATRA treatment, leiomyoma cells demonstrated a down-regulation of fibronectin gene to levels comparable to myometrial expression patterns.

**Fig. 4:**
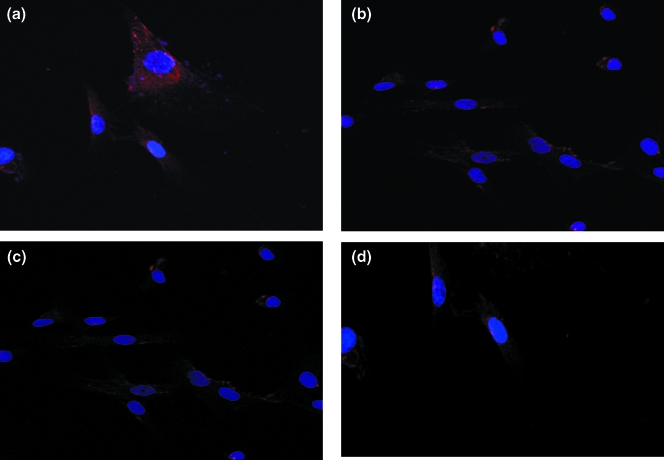
Analysis of fibronectin protein in myometrium and leiomyoma cells as analysed by cytoimmunofluorescence. Pre-treatment leiomyoma cells (a) demonstrated a higher amount of protein (higher red fluorescence) compared to myometrial cells (c). Decreased red fluorescence in post ATRA (10^−7^m) treated leiomyoma cells (b) indicated a decreased amount of fibronectin protein. Minimal decreased red fluorescence was observed in myometrial cells (d). Red fluorescence is indicative of fibronectin protein, DAPI (blue fluorescence) strongly binds to DNA and indicates the nucleus in the cell. Magnification: 40×.

In order to confirm that mRNA alterations translated into protein alterations, we used cytoimmunofluorescence to determine the production of fibronectin protein in leiomyoma and myometrial cells in culture. In untreated leiomyoma cells, we demonstrated a higher amount of fibronectin protein in leiomyoma compared with myometrial cells (Fig. 4). Post-treatment with 10^−7^m ATRA, the leiomyoma cells (Fig. 4b) reduced fibronectin protein. Again, fibronectin protein production in the presence of ATRA was comparable between leiomyoma and myometrial cells.

### Versican variants respond to ATRA treatment

Versican is an ECM proteoglycan that represses adhesion between cells and plays a major role in many cellular processes in which tissue remodelling of ECM is central.^17^ Increased versican expression is observed during pregnancy in cervix,^18^ suggesting that increased versican may characterize a loosely organized ECM. Dysregulated, larger and loosely arranged ECM is a hallmark of leiomyomas compared to surrounding myometrium.^3^,^16^ We have previously shown increased versican expression in leiomyoma cells compared to myometrial cells in culture.^8^ Based on the effect of ATRA on collagens and other ECM proteins, we hypothesized that ATRA exposure would result in down-regulation of versican.

There are four isoforms of versican, V0, V1, V2 and V3, arising due to differential gene splicing.^17^,^19^ We demonstrated a 7·75-fold increase (± 2·2; *P* < 0·05) of versican V0 mRNA expression in leiomyoma cells as compared to myometrial cells (Table 1). There was a marked decrease of versican V0 expression at 10^−7^m ATRA, and by 10^−6^m ATRA the expression of V0 in leiomyoma and myometrial cells was essentially at a 1 : 1 ratio (Table 1).

Similarly, versican V1 mRNA was 4·6 ± 0·45-fold elevated and variant V3 was 8·32 ± 1·21-fold elevated in leiomyomas as compared to myometrium in absence of ATRA exposure (Table 1). On exposure to ATRA concentrations of 10^−8^m and greater, variant V1 was down-regulated (0·71 ± 0·13; *P* < 0·05) in leiomyomas. Similarly, V3 demonstrated a down-regulation of the mRNA expression in leiomyoma cells as compared to myometrial cells on exposure to ATRA (Table 1). Versican variant V2 did not demonstrate statistically significant difference in untreated leiomyoma and myometrial cells, although ATRA treatment down-regulated the mRNA expression of this variant as well (data not shown).

**Fig. 5:**
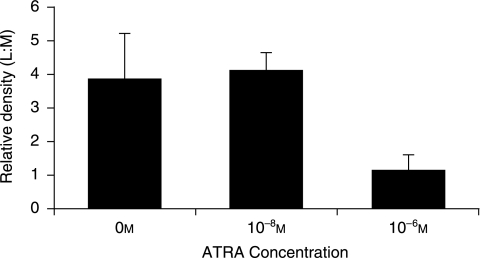
ATRA treatment reduced the amount of versican protein in leiomyoma cells to an equivalent level of expression as myometrial cells. The fold difference between leiomyoma and myometrial cells (L : M) is based on differences in relative density measured using QualityOne software (Bio-Rad). The relative density units was corrected for internal control, β-actin.

To determine if changes observed at V0/V1 mRNA level translated into changes in versican protein production, we used Western blot analysis. As seen in Fig. 5, ATRA reduced the concentration of versican protein in myometrial and leiomyoma cells. In untreated cells, approximately fourfold higher protein concentration was seen in leiomyoma cells as compared to myometrial cells. At ATRA concentration of 10^−6^m, the amount of protein in leiomyoma was close to the amount found in myometrium cells (1·14 ± 0·51-fold; Fig. 5).

### TGF-β3 gene expression is affected by ATRA treatment

Interaction between RA and the TGF-β pathway has been demonstrated in different cell types, including Wilms tumour cells,^20^ lung cancer cells^21^ and mesenchymal cells C3H10T1/2.^22^ TGF-β pathway also plays a major role in leiomyogenesis.^23^,^24^ Expressions of all TGF-β isoforms and receptors have been reported in both leiomyomas and myometrium.^25^–^28^ We and other laboratories have previously demonstrated an increased expression of TGF-β3 in leiomyoma compared to myometrium.^8^,^23^,^25^,^27^,^29^,^30^ The TGF-β pathway is also known to play a role in ECM remodelling in various tissues.^31^ We therefore hypothesized that leiomyoma cells exposed to ATRA would demonstrate lower expression of TGF-β3 resulting in modulation of TGF-β pathway as well as the TGF-β regulated genes.

In these studies, we have demonstrated that TGF-β3 mRNA was significantly elevated in untreated leiomyoma cells as compared to myometrial cells (2·96 ± 0·92-fold; *P* < 0·05). We observed a down-regulation of TGF-β3 mRNA in leiomyoma on exposure to ATRA. As shown in Table 1, on exposure to 10^−8^m ATRA concentration, the expression of TGF-β3 mRNA in leiomyoma cells was similar to myometrial cells (0·84 ± 0·12; *P* < 0·05), indicating that ATRA down-regulated the TGF-β3 expression and thus may indirectly affect the TGF-β pathway.

To determine whether ATRA impacted the TGF-β pathway regulation of specific genes, we examined the expression of matrix metalloproteinase-2 (MMP-2) which is regulated by TGF-β in leiomyomas.^32^,^33^ As shown in Table 1, untreated leiomyoma cells demonstrated a 6·65 ± 1·22-fold elevation of MMP-2 gene. At 10^−8^m ATRA, down-regulation of MMP-2 was observed, resulting in comparable expression in both cell lines. These results suggest that ATRA may also affect the excessive and disorganized ECM expression through the TGF-β pathway.

## Discussion

Our results demonstrated that a rise in ATRA exposure impacted leiomyoma cell proliferation and altered ECM production. Low concentrations of ATRA (10^−8^m) inhibited growth of leiomyoma cells in culture and regulated expression of leiomyoma cell ECM structural components, proteoglycans, and cytokines to levels that approximated expression patterns of myometrial cells. These results suggest that the metabolic profile identified in human leiomyomas *in-situ* resulting in reduced exposure to active RA metabolites may be critical in maintaining the leiomyoma phenotype.

The data from various laboratories on the impact of RA on leiomyoma proliferation has been controversial. RA has been shown to induce leiomyoma development in animal models for leiomyomata.^30^,^34^ Tsibris and coworkers^30^ demonstrated that ATRA as well as 9-*cis*-RA in the presence of E_2_-induced uterine leiomyoma tumours. Using the Eker rat leiomyoma model, a genetic model generated by the disruption *tuberous sclerosis 2* gene expression, Gamage and coworkers^34^ demonstrated that use of retinoid X receptor-selective agonist, LGD 1069, reduced the number of grossly observable tumours due to increased apoptosis, but not the total incidence, suggesting that RA agonists can inhibit leiomyoma growth. In contrast, *in vitro* studies in primary cultures derived from human leiomyoma suggested that augmentation of RA exposure led to inhibition of cell growth.^35^,^36^ Boetter-Tong and colleagues found that ATRA inhibited leiomyoma and myometrial cell proliferation in a reversible manner.^37^ When these primary cell lines were treated with RA, Mangioni demonstrated an inhibition of Wnt5b mRNA production.^35^ Retinoid analogue 4-(*N*-hydroxyphenyl)retinamide (4-HPR) inhibited growth and induced apoptosis in primary cultures derived from uterine leiomyomas without affecting myometrial cell growth.^36^ Given this controversy, and the limitations of primary cultures, we generated novel immortalized and molecularly confirmed leiomyoma and myometrial cell lines to evaluate the impact of RA.

In our study, at least a 10-fold higher concentration of ATRA was required to observe significant growth inhibition in myometrial cells. ATRA at 10^−8^m is within the physiological dose^38^ that has been observed for various cellular activities including embryogenesis and differentiation of different cell types, indicating that leiomyoma tumours may respond to physiological and pharmacologically relevant doses^38^ of ATRA *in vivo*.

We had previously demonstrated that the rate of metabolism of ATRA and 9-*cis*-RA was higher in leiomyoma tissue compared to myometrium tissue.^6^ Therefore, it was unexpected that growth of leiomyoma cells responded to ATRA concentrations lower than those required to see an effect in myometrial cells. Given the increased activity of metabolizing enzyme CYP26A1 in leiomyoma cells,^8^ we expected that there would be greater metabolism of active ATRA compound, thus negating the retinoid effect. Various investigators have found a dose-dependent up-regulation of CYP26A1 by ATRA in different cell types.^12^,^13^ In leukaemia cells, ATRA can induce CYP26A1 at doses of 1 µm,^12^ whereas in endothelial cells, 10 µm ATRA was required to induce expression of CYP26A1.^13^ Our results demonstrated that CYP26A1 transcripts were up-regulated in myometrial cells exposed to ATRA when compared to unexposed cells. In contrast, slight down-regulation of CYP26A1 was observed in ATRA exposed leiomyoma cells when compared to unexposed control cells. Significant up-regulation of CYP26A1 in myometrial cells and down-regulation in leiomyoma cells resulted in approximate equal levels of CYP26A1 in these cells at 10^−6^m ATRA concentration.

Leiomyomas share phenotypic characteristics with normal myometrium and fibroblasts. While speculative, it is possible that leiomyoma formation results from myometrial cells that undergo altered differentiation that results in a phenotype defined by excessive and disorganized ECM production.^3^ Retinoids are regulators of differentiation in many cell types. In cultured stellate cells, it has been demonstrated that ATRA can prevent morphological transition towards a myofibroblast phenotype and decrease collagen 1 synthesis.^37^ In addition, ATRA can inhibit radiation-induced pulmonary fibrosis.^39^ The authors report inhibition of COL1A1 mRNA expression by ATRA in irradiated lung tissue. They further propose a role for TGF-β pathway in pulmonary fibrosis that is inhibited by ATRA. Finally, redifferentiation, ECM synthesis and degradation is regulated by ATRA in human arterial smooth muscle cells.^40^

The mechanism of ATRA in leiomyoma cells that results in ECM regulation remain speculative. ATRA is known to act through different pathways including MAP kinase pathway^39^,^41^,^42^ and protein kinase C pathway.^39^,^43^ In this study, we demonstrated an antifibrotic potential of ATRA on overall production of collagens secreted into the ECM as well as the mRNA of collagen types 1, 3 and 4, fibronectin and versican in leiomyoma cells. At a dose of 10^−8^m ATRA, we observed decrease in production of soluble collagens, indicating that there may be changes in overall ECM production by leiomyoma cells.

TGF-β3 plays an important role in ECM accumulation. It is known as an inducer of ECM proteins and its role in the pathogenesis of fibrosis is well established. Up-regulation of ECM deposition by TGF-β pathway can take place through different mechanisms, including stimulation of collagen synthesis and regulation of MMPs.^31^,^32^ Altered expression of TGF-β plays an important role in leiomyogenesis^7^,^25^–^29^ as demonstrated *in vivo* and *in vitro*. In leiomyomas, TGF-β regulation of ECM proteins such as collagens^23^ and fibronectin,^26^,^27^,^29^ and has also been demonstrated. Regulation of TGF-β by retinoids has been demonstrated in various processes such as chondrogenesis^44^,^45^ and morphogenesis of the mammary gland.^12^ We have demonstrated an interaction between ATRA and the TGF-β pathway in leiomyomas by demonstrating ATRA down-regulation of TGF-β3 expression and expression of TGF-β regulated genes such as the MMPs. We further hypothesize that both these pathways may play a synergistic role in inducing the abnormal ECM remodelling in leiomyogenesis and could potentially play a significant role clinically as combinational therapy for treatment for leiomyomas.

In summary, we have found that ATRA significantly reduced growth and synthesis of extracellular matrix proteins in leiomyoma cells, and may exert some of its effect by regulating the TGF-β signalling pathway.
